# Supplementary material for: Designing Stable Bacillus anthracis Antigens with a View to Recombinant Anthrax Vaccine Development
Source: Pharmaceutics. 2022 Apr 6;14(4):806. doi: 10.3390/pharmaceutics14040806 (PMC9025368; doi:10.3390/pharmaceutics14040806)
Supplement: Supplementary file 1 [file pharmaceutics-14-00806-s001.zip › pharmaceutics-1634389-supplementary.pdf]

pharmaceutics

MDPI

Supplementary Materials: Designing Stable *Bacillus anthracis* Antigens with a View to Recombinant Anthrax Vaccine Development

Ekaterina M. Ryabchevskaya, Dmitriy L. Granovskiy, Ekaterina A. Evtushenko, Peter A. Ivanov, Olga A. Kondakova, Nikolai A. Nikitin and Olga V. Karpova

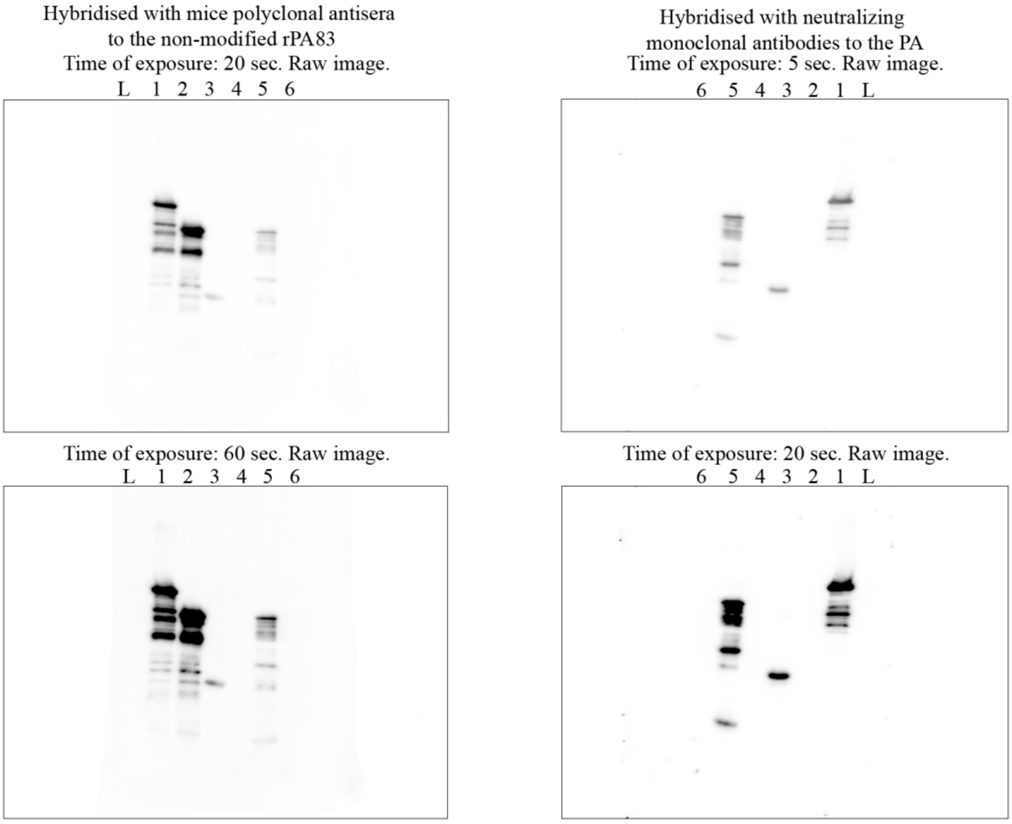

Figure S1. Original images of the western blot membranes represented in Figure 3.

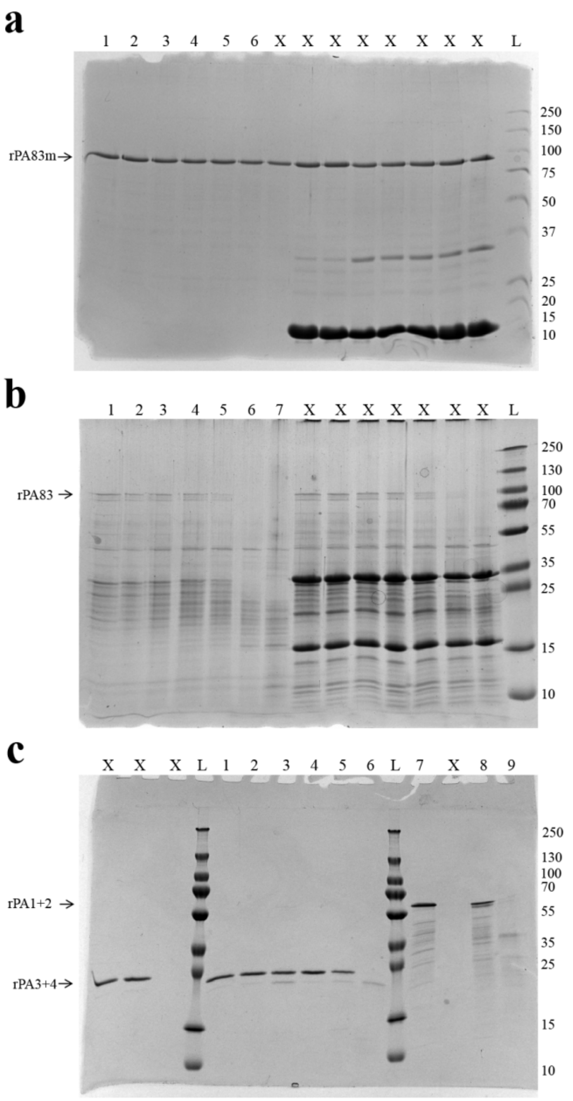

Figure S2. Original images of the gels represented in Figure 4.

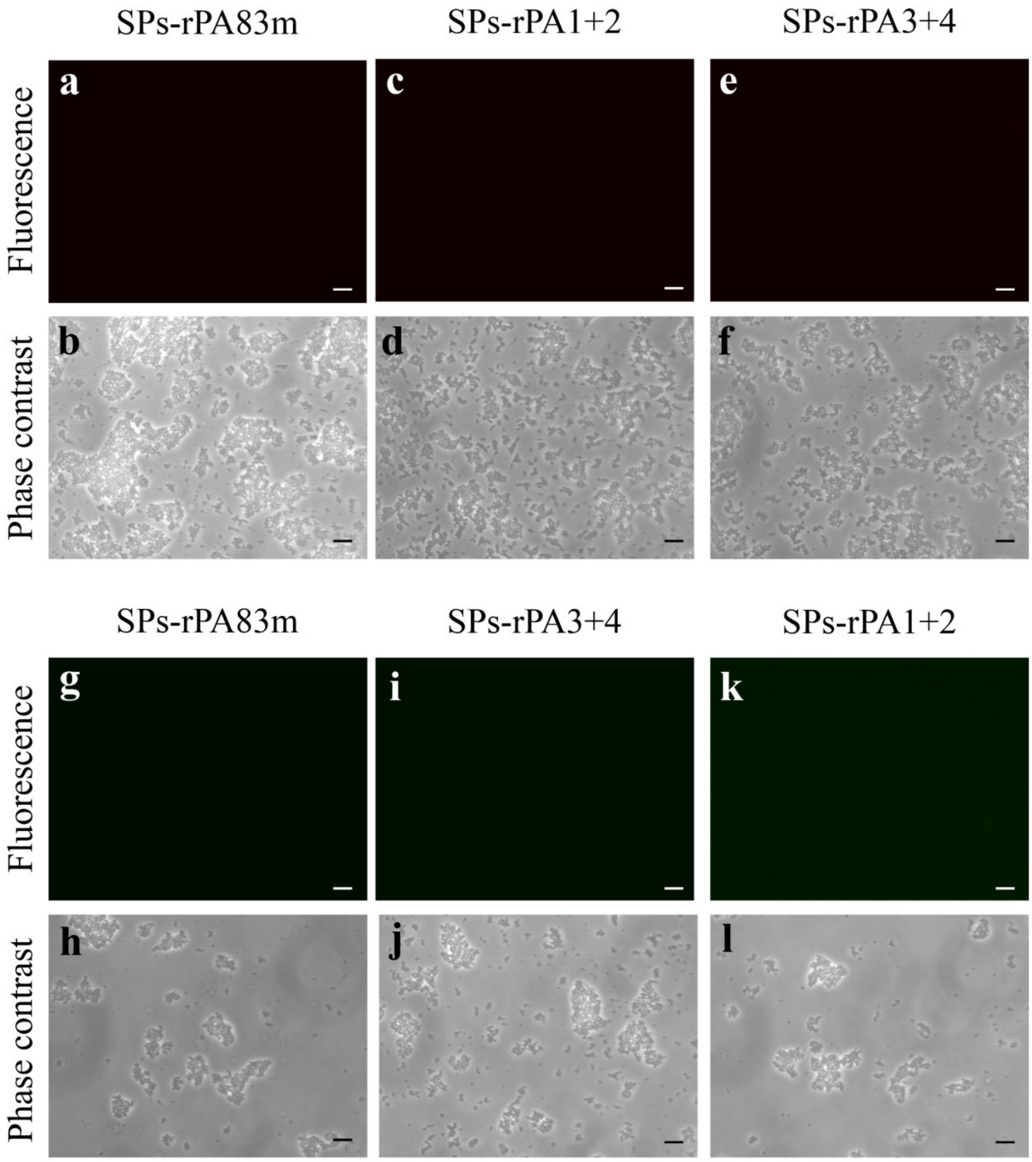

Figure S3. Images of the negative control samples without primary antibodies for the fluorescence analysis represented in the Figure 5 (a–j), and negative control (k—fluorescence analysis, l—phase contrast) represented by SPs-rPA1+2 compositions treated with chimeric monoclonal PA-neutralising antibodies and secondary antibodies conjugated to CF™ 488A.

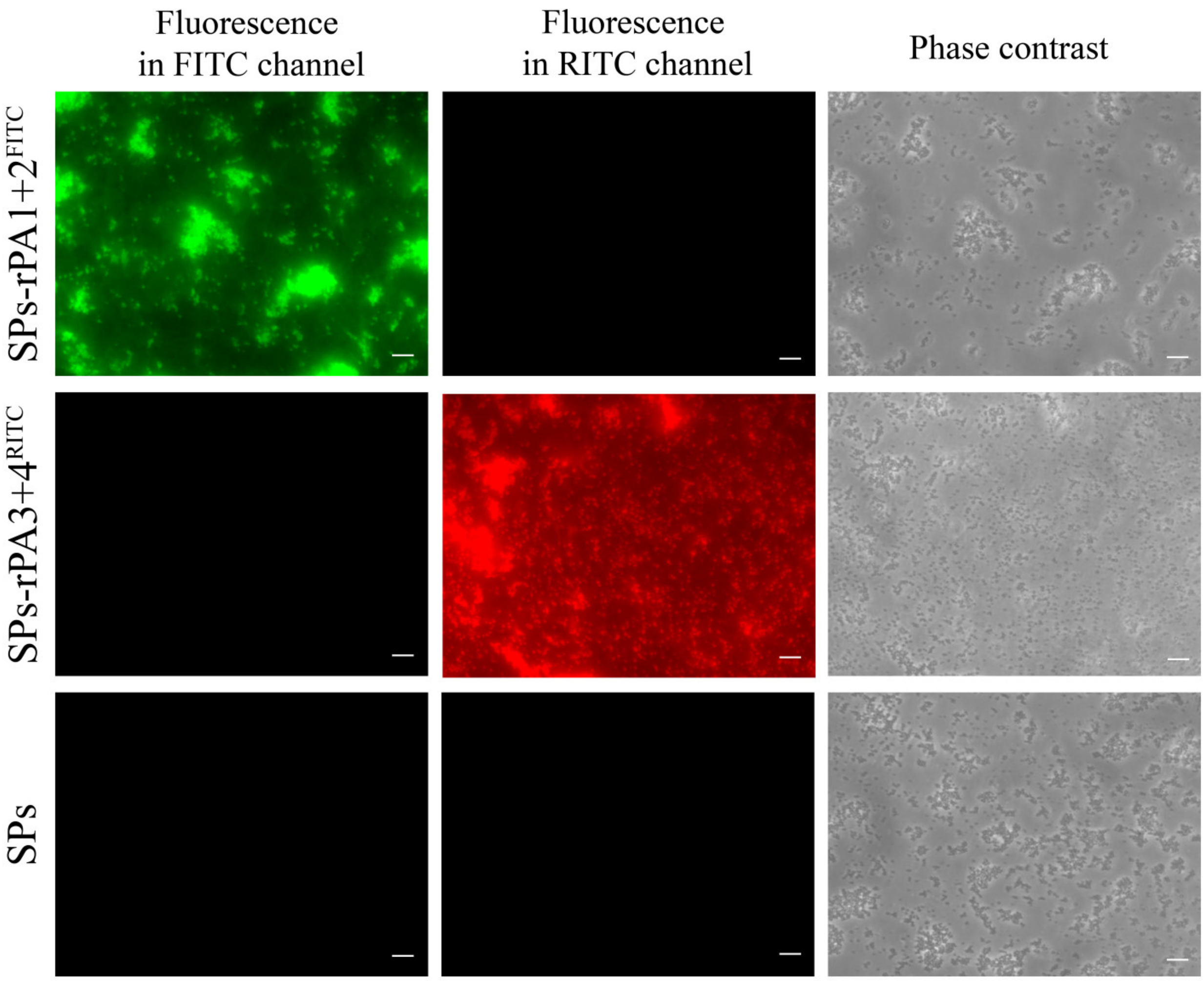

Figure S4. Images of the control samples for the fluorescence analysis represented in the Figure 6a–c.

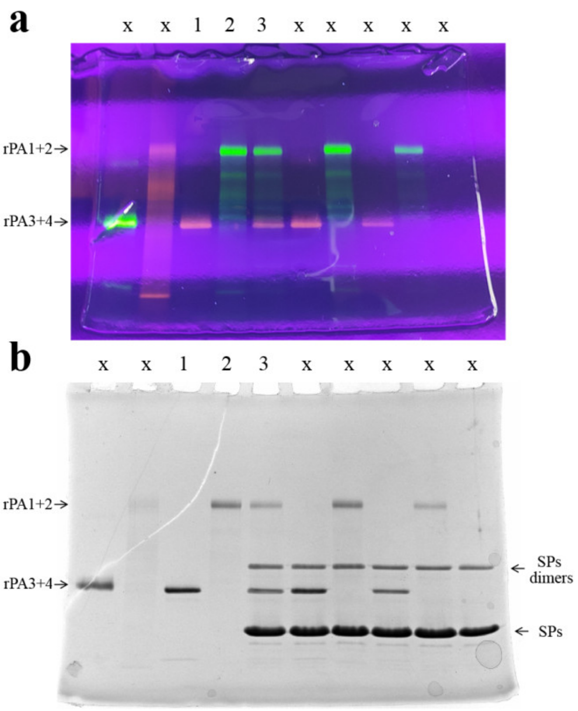

Figure S5. Original images of the gels represented in Figure 6d–e.

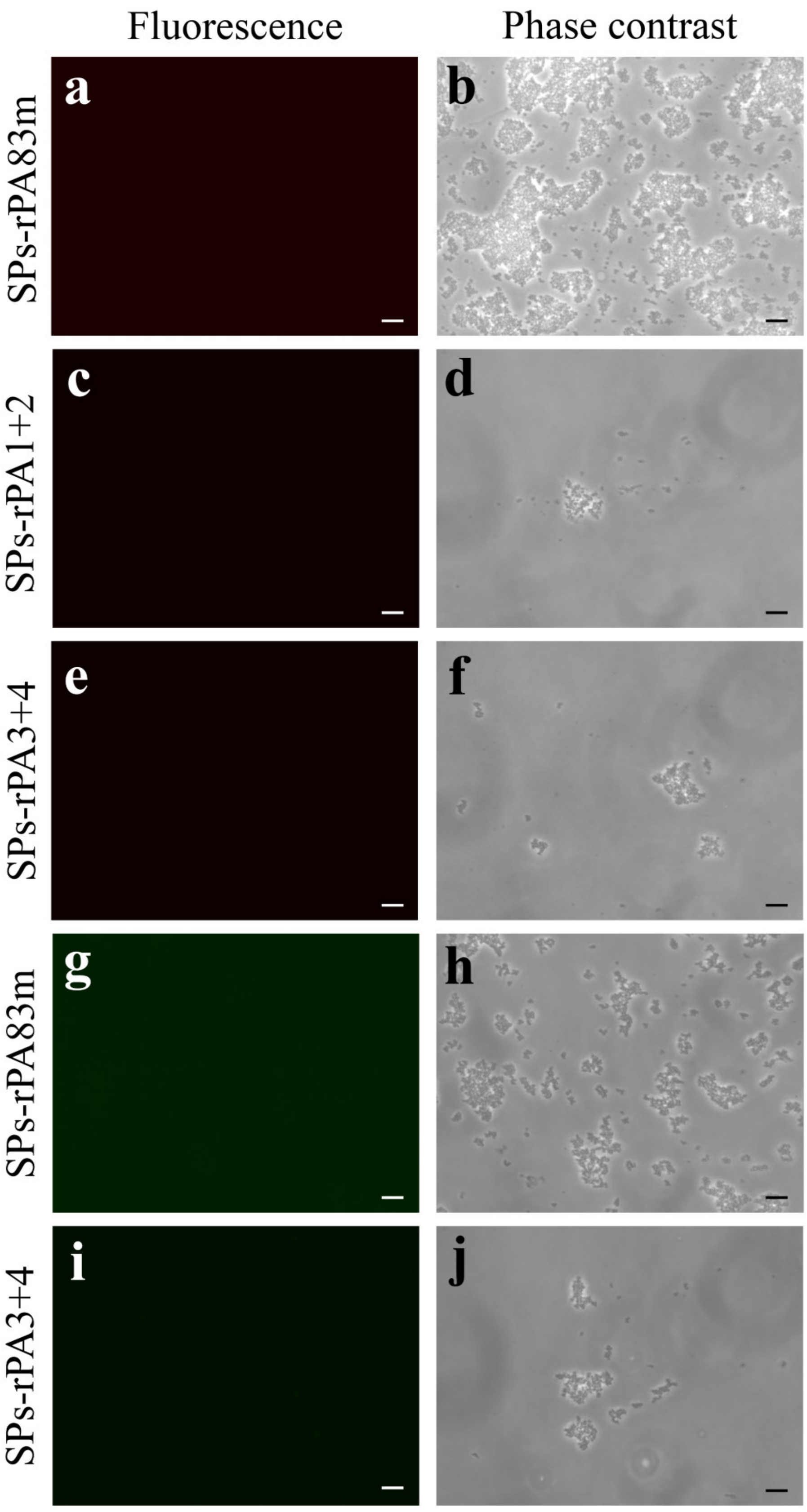

Figure S6. Images of the negative control samples without primary antibodies for the fluorescence analysis represented in the Figure 8.
